# Higher primate-like direct corticomotoneuronal connections are transiently formed in a juvenile subprimate mammal

**DOI:** 10.1038/s41598-018-34961-z

**Published:** 2018-11-08

**Authors:** Naoyuki Murabe, Takuma Mori, Satoshi Fukuda, Noriko Isoo, Takae Ohno, Hiroaki Mizukami, Keiya Ozawa, Yumiko Yoshimura, Masaki Sakurai

**Affiliations:** 1 Department of Physiology, Teikyo University School of Medicine, Tokyo, 173-8605 Japan; 2 Division of Visual Information Processing, National Institute for Physiological Sciences, National Institutes for Natural Sciences, Okazaki, 444-8585 Japan; 3 Division of Genetic Therapeutics, Jichi Medical University, Tochigi, 329-0498 Japan; 4 Research Hospital, Institute of Medical Science, Tokyo University, Tokyo, 108-8639 Japan; 5 Department of Physiological Sciences, Graduate University for Advanced Studies, Okazaki, 444-8585 Japan; 6 Department of Molecular and Cellular Physiology, Institute of Medicine, Academic Assembly, Shinshu University, Nagano, 390-8621 Japan

## Abstract

The corticospinal (CS) tract emerged and evolved in mammals, and is essentially involved in voluntary movement. Over its phylogenesis, CS innervation gradually invaded to the ventral spinal cord, eventually making direct connections with spinal motoneurons (MNs) in higher primates. Despite its importance, our knowledge of the origin of the direct CS-MN connections is limited; in fact, there is controversy as to whether these connections occur in subprimate mammals, such as rodents. Here we studied the retrograde transsynaptic connection between cortical neurons and MNs in mice by labeling the cells with recombinant rabies virus. On postnatal day 14 (P14), we found that CS neurons make direct connections with cervical MNs innervating the forearm muscles. Direct connections were also detected electrophysiologically in whole cell recordings from identified MNs retrogradely-labeled from their target muscles and optogenetic CS stimulation. In contrast, few, if any, lumbar MNs innervating hindlimbs showed direct connections on P18. Moreover, the direct CS-MN connections observed on P14 were later eliminated. The transient CS-MN cells were distributed predominantly in the M1 and S1 areas. These findings provide insight into the ontogeny and phylogeny of the CS projection and appear to settle the controversy about direct CS-MN connections in subprimate mammals.

## Introduction

The corticospinal (CS) tract is a phylogenetically novel descending pathway that emerged and evolved in mammals, culminating in the higher primates, and is centrally involved in voluntary movements^1,2^. Voluntary movement is historically thought to be achieved by associating, integrating and modifying the functions of lower-level circuits, including reflex arcs and central pattern generators, implemented in the spinal cord and brain stem^3^. In higher primates, however, a considerable portion of CS axons bypass phylogenetically older circuits and make direct connections with spinal motoneurons (MNs), the “final common path”^3^ of the central nervous system, and this is believed to provide the basis for the manual dexterity seen in primates^4–7^. Our knowledge about the phylogenetic origin of this direct CS-MN connection is limited, however, and there is controversy as to whether these direct CS-MN connections are also present in lower, subprimate mammals^8–11^, including rodents. We previously found that CS axons make direct synapses on MNs innervating distal forelimb muscles at an early stage of development in rats^12^ and mice^13^, which was also shown by a different group, recently^14^. Whether these direct CS-MN connections are transient or persist into adulthood is not yet clear, however. The fact that spinal cord slices with adequate viability, especially among MNs, are available only up to the second postnatal week^15–17^ has hampered rigorous electrophysiological examination of this question. Moreover, it is not easy to demonstrate the absence, instead of the presence, of CS-MN connections from recordings of single MNs, though population studies using rabies virus could be an excellent method for this purpose, as they would reveal the spatial distribution of CS neurons connecting to MNs.

In this study, we addressed this problem in mice using retrograde monosynaptic tracing methods with genetically-modified rabies viruses^18,19^. Rabies virus infects neurons and is retrogradely transported across synapses, which enables its use as a retrograde transsynaptic tracer^20,21^. Callaway and colleagues recently developed a method for retrograde monosynaptic tracing using a genetically modified rabies virus in which the gene encoding an envelope glycoprotein (G protein) essential for neuronal infection is replaced by the gene encoding GFP^18^. For initial infection of first-order neurons, one type of the virus possessing the native rabies G protein in their envelope is employed. Another way is to produce a virus that carries an avian virus envelope protein (EnvA) instead of G protein, which specifically binds to tumor virus receptor subgroup A (TVA). TVA is a receptor for EnvA and is not encoded in the mammalian genome. This allows the rabies to specifically infect only TVA-expressing neurons. Both virus types are able to spread from first- to second-order neurons only when G protein is complemented within the first-order neurons.

We found that CS neurons make direct connections with cervical MNs innervating forearm muscles, but not lumbar MNs innervating hindlimbs, and that these direct CS-MN connections are present at an early stage of development, but are later eliminated during development. These transient corticomotoneuronal (CM) cells predominantly were distributed in the M1 and S1 areas. These findings appear to settle the long-standing controversy about the direct CS-MN connections in subprimate mammals; moreover, they provide insight into the ontogeny and phylogeny of the CS projection.

## Results

### Direct corticospinal connections with the MNs innervating the forearm muscles in juvenile mice

**Figure 1:**
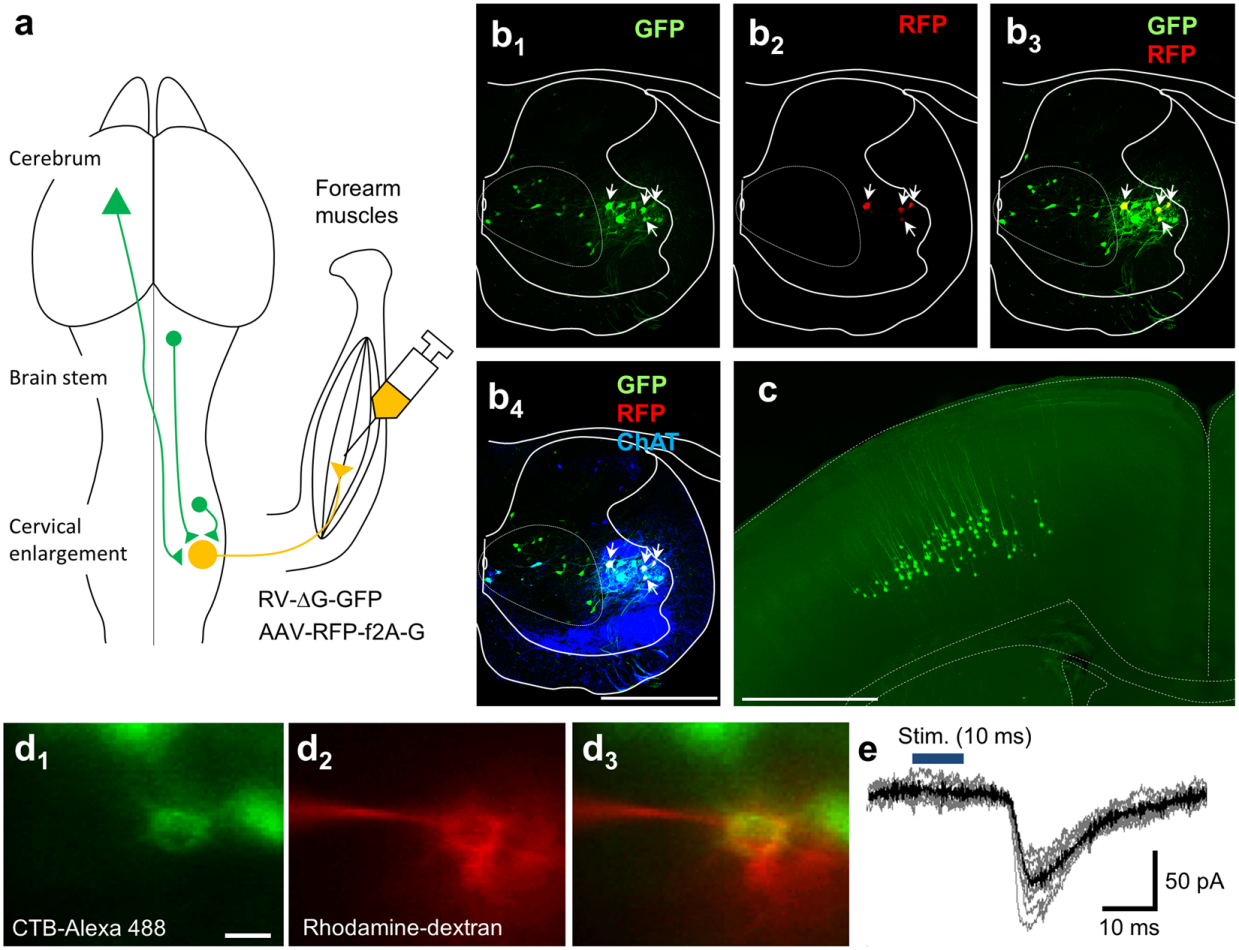
CS neurons made monosynaptic connections with forearm MNs in P14 animals. (**a**) Experimental design for retrograde monosynaptic tracing from forearm MNs. RV-ΔG-GFP and AAV6-RFP-f2A-G were co-injected into forearm muscles. Both viruses infect the MNs from the terminals and are retrogradely transported to the somata. RV-ΔG-GFP spreads into presynaptic cells (green) through complementation of G protein encoded by AAV. (**b**) Infection by RV-ΔG-GFP and AAV6-RFP-f2A-G of forearm MNs revealed by expression of GFP from RV-ΔG-GFP and RFP from AAV6-RFP-f2A-G. Cells in which both fluorescent proteins were co-expressed are starter neurons for monosynaptic tracing (arrows). GFP-labeled neurons in lamina V are shown inside the dotted line. b1, GFP; b2 RFP; b3, merged image (GFP, RFP) and b4, merged image (GFP, RFP, anti-choline acetyl transferase (ChAT) immunostaining). Animals were received intramuscular injection on P7 and sacrificed on P14. The images are representative of three independent experiments. (**c**) GFP-positive cells in layer V of the cerebral cortex. Superimposed view from 10 sections. Animals were received intramuscular injection of RV-ΔG-GFP and AAV6-RFP-f2A-G on P5 and sacrificed on P14. The image is representative of five independent experiments. (**d1**) Forearm MNs identified on P10 as cells retrogradely labeled with CTB-Alexa 488 injected intramuscularly on P7. (**d2**) A targeted CTB-Alexa488-positive cell into which rhodamine-dextran was perfused through a whole-cell pipette. (**d3**) Merged image of d1 and d2 certifying that whole cell recordings were made from a forearm MN. The images are representative of thirteen independent experiments. (**e**) EPSCs recorded from a forearm MN in the presence of high concentrations of divalent cations (7 mM Ca^2+^, 3 mM Mg^2+^) and showing a fixed latency in response to optogenetic stimulation (blue bar) of CS axons. Scale bars indicate 500 μm in (**b)**, 1 mm in (**c)** and 20 μm in (**d)**. The traces are representative of seven independent experiments.

A mixture of G protein-deleted rabies virus expressing GFP (RV-ΔG-GFP), which is incompetent for transsynaptic spread^18,19,22^ and adeno-associated virus (AAV) serotype 6 encoding the G protein and RFP, which also lacks ability of transsynaptic spread alone was injected into the forearm muscles of mice on postnatal day (P) 5 to P7 (Fig. 1a). For effective viral infection of forearm MNs, the injections were guided by a self-made map of the end plates of the forearm muscles visualized using acetylcholinesterase histochemistry (Supplementary Fig. S1)^23^. Since spinal cord neurons extending axons to skeletal muscles are only MNs, initial infection after intramuscular injection of viruses are limited to MNs in the spinal cord neurons^19,22,24^, which is an advantage for monosynaptic tracing from MNs in juvenile animals. Because the G protein gene is carried by an AAV, only MNs infected with both viruses, referred to as starter MNs, enable the rabies virus to transport retrogradely across synapses into their premotor neurons. Within premotor neurons, where G protein is no longer available, RV-ΔG-GFP ceases to propagate and thus reveals the premotor neuron distribution. We confirmed that RV-ΔG-GFP did not spread to premotor neurons when MNs was infected with RV-ΔG-GFP alone, (Supplementary Fig. S2).

Starter MNs, coexpressing GFP, RFP and a MN marker, choline acetyl transferase, were found in the dorsal part of the ventral horn (Fig. 1b), where the forearm MN pools are located^25^, from the C5 to Th1 spinal cord^23^ 7 days after intramuscular injections of the viruses. Transsynaptically labeled neurons (i.e. premotor neurons), expressing GFP but not RFP (Fig. 1b, dotted line) were abundantly found in intermediate zone including lamina V, which is consistent with previous report^26^. Premotor neurons were found along the cervical cord and brain stem, including the vestibular nucleus and reticular nuclei as well as the red nucleus (Supplementary Fig. S3). We also found labeled neurons in the cerebral cortex 8–9 days after intramuscular virus injections (Fig. 1c and Supplementary Fig. S4) while degeneration of starter MNs subsequently proceeded^27^, which prevented further examination of the starter MNs. Premotor neurons in the cortex were located in layer V, possessed apical dendrites and expressed CTIP2, a molecular marker for the CS neurons^28^, indicating features typical of CS neurons (Fig. 1c and Supplementary Fig. S4). This result indicates the presence of monosynaptic connections between CS neurons and forearm MNs and these labeled cells in the cortex represent corticomotoneuronal cells.

To confirm the direct CS-MN connections electrophysiologically, we stimulated CS axons and recorded electrical responses from MNs in spinal cord slices. For selective stimulation of CS axons, we injected AAV1 encoding channelrhodopsin-2-EYFP into the sensorimotor cortex of P0 pups. To identify forearm MNs, we used retrograde labeling with cholera toxin B subunit-conjugated Alexa 488 through intramuscular injections (Supplementary Fig. S5), which is a standard procedure for identification of MNs^29–34^. On P8-P10, slices were prepared from the caudal cervical cord, and whole cell recordings were made from labeled cells (Fig. 1d). We used the following established criteria to define monosynaptic excitatory postsynaptic currents (EPSCs): i) the EPSC onsets had fixed latencies^12,35–37^; ii) the recorded EPSCs consistently followed the same time course, including the time to peak and decay time, without failure^12,35,38,39^; and iii) the responses were recorded in the presence of high concentrations of divalent cations (7 mM Ca^2+^, 3 mM Mg^2+^) that preferentially abolishes polysynaptic responses by increasing the threshold for spike generation^12,39–42^. Under these criteria light activation of CS axons elicited monosynaptic EPSCs in approximately 50% (6/13) of forearm MNs (4.9 ± 1.2 ms in latency, 142.2 ± 55.8 pA in amplitude, mean ± SEM). (Fig. 1e).

### Distribution of the corticomotoneuronal cells in the juvenile mouse cortex

**Figure 2:**
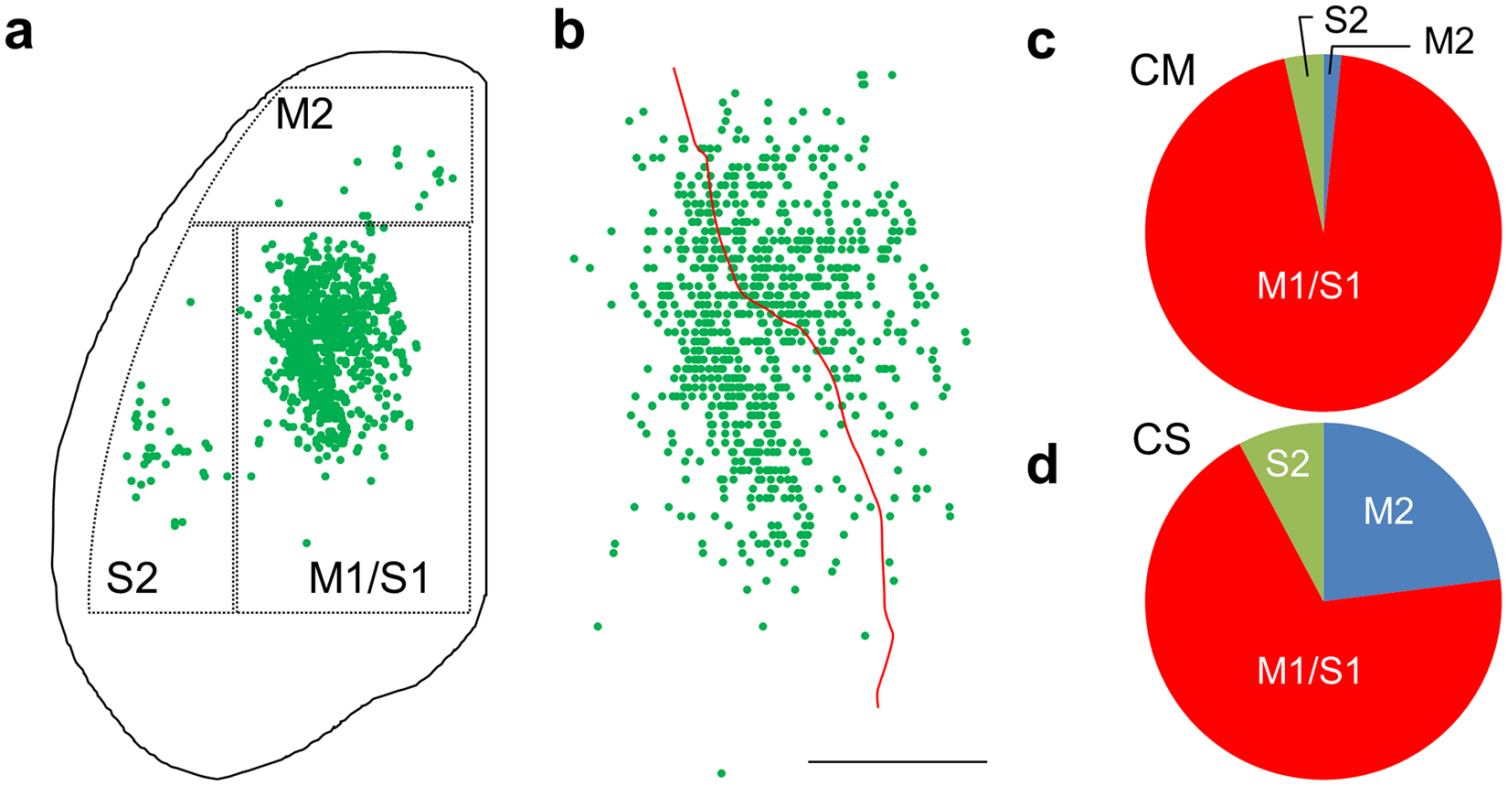
CM cell distribution. (**a**) Distribution of CM cells in the contralateral cortex of P14 mice (data from 5 mice were superimposed). (**b**) Distribution of CM cells in M1/S1 areas. The red curve indicates the boundary between M1 and S1. (data from 5 mice were superimposed). (**c**) Proportion of CM cells located in the M1/S1 areas. (n = 5) Animals were received intramuscular injection of RV-ΔG-GFP and AAV6-RFP-f2A-G on P7 and sacrificed on P14 (**a**–**c**). (**d**) Proportion of C7 projecting CS neurons. (n = 10). Data were used from previous experiment^43^. Scale bars indicate 2 mm in a and 1 mm in b.

To ask whether CS neurons innervating caudal cervical cord in any cortical region made direct connections onto motoneurons in juveniles or there are hot spots in which CM cells reside, we next mapped the locations of the cells labeled by rabies virus in the cerebral cortex (Fig. 2a,b and Supplementary Fig. S6). The labeled cells, i.e. CM cells, were distributed in the rostral approximately two-thirds of the contralateral hemisphere. A small number of CM cells were also found in the sensorimotor area of the ipsilateral cortex (Supplementary Fig. S6), which accounted for 2.0 ± 0.7% (n = 5) of the total labeled cortical neurons. The CM cells in the contralateral cortex were clustered around three regions: a caudal part, including the primary motor and primary somatosensory areas (M1/S1); a rostral part, including the secondary motor area (M2); and a caudolateral part, including the secondary somatosensory areas (S2). We calculated the proportion of CM cells locating in M1/S1, M2 and S2 regions, respectively. The CM cells were predominantly located in M1/S1 areas (95 ± 1%) (Fig. 2c). Within M1/S1, the labeled cells were present almost equally in the two areas (48 ± 4% in M1; 52 ± 4% in S1). The labeled cells in the M2 (2 ± 1%) and S2 (4 ± 1%) areas represented minor populations. These results contrasted with our previous finding that the CS neurons projecting to the cervical enlargement (C7) are widely distributed in juvenile cortex (Fig. 2d), which was studied by injection of a retrograde tracer into the C7 to cover >80% of the gray matter^43^. It is thus suggested that CS neurons in specific areas corresponding to M1/S1 region preferentially make direct connections with the forearm MNs.

### Monosynaptic tracing from MNs innervating lower-leg muscles in the juveniles

**Figure 3:**
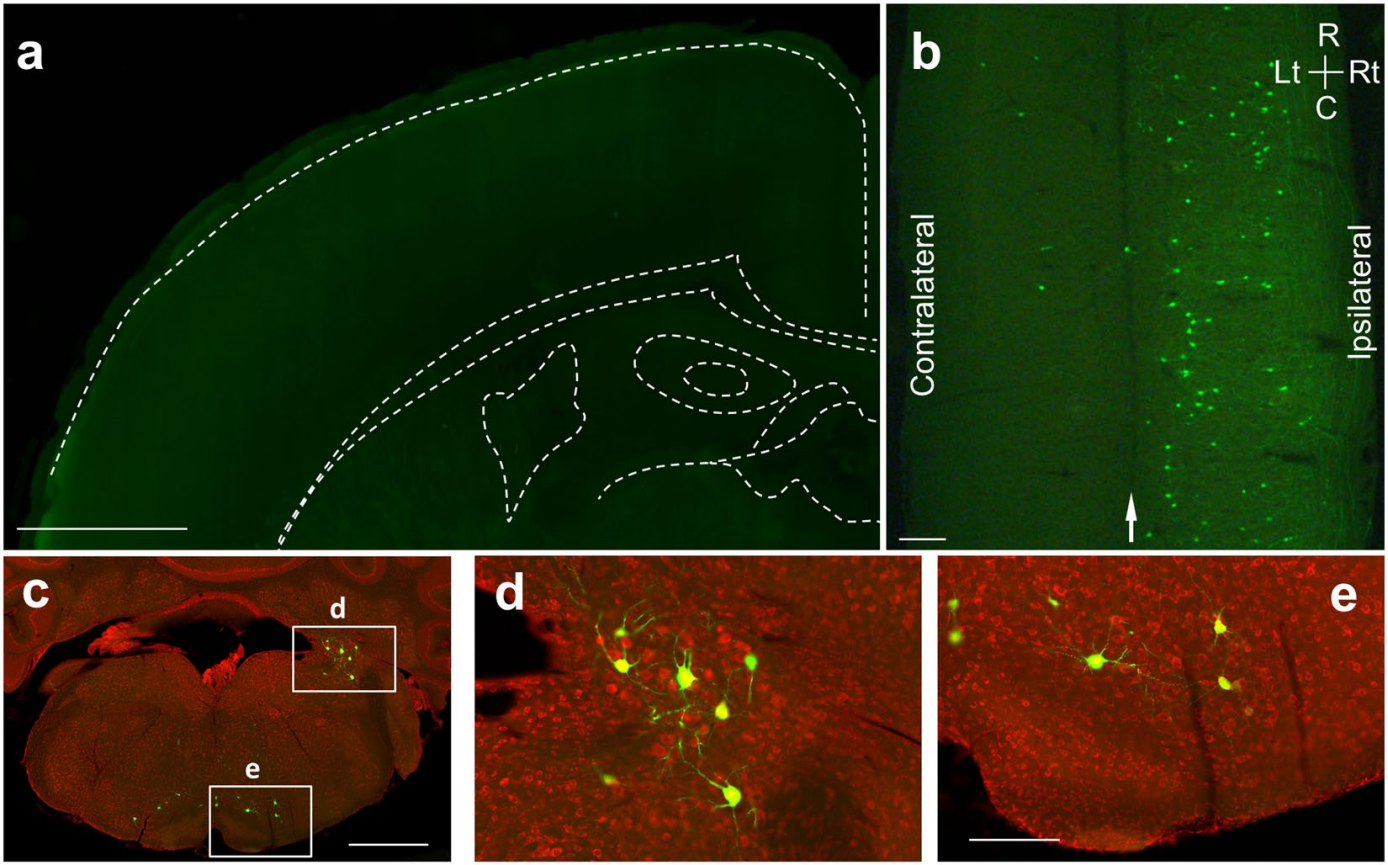
Monosynaptic tracing from lower-leg MNs showed an absence of CM cells. (**a**) Example of the contralateral cortex showing the absence of the CM cells. Superimposed view from 10 sections. Scale bar, 1 mm. (**b**) Longitudinal section of the lumbar spinal cord showing GFP-labeled interneurons. C, caudal, R, rostral, Lt, left, Rt, right. Arrow, midline. Scale bar, 200 μm. (**c**) Coronal section of the medulla oblongata showing GFP-labeled neurons. Scale bar, 1 mm. (**d,e**) High power views of selected regions in **c** showing GFP-labeled neurons in the vestibular nucleus (**d**) and reticular formation (**e**). Scale bar, 250 μm. Red, a fluorescent Nissl staining by Neurotrace Red. Animals were received intramuscular injection on P8 and sacrificed on P18. The images are representative of five independent experiments.

We also tested whether lumbar MNs of juvenile mice received direct synaptic connections from CS neurons. CS axons reach the gray matter of the lumbar cord around P14, which is about 7 days later than the cervical cord^44^, and form functional synapses with interneurons in the L4 on P14^43^. Hence, we injected RV-ΔG-GFP and the AAVs encoding G protein and RFP into the lower-leg (distal prat of the hindlimb, below the knee joint) muscles on P8. Six days after injections, we found transsynaptically labeled neurons in the lumbar spinal cord as well as MNs, indicating that premotor neurons were labeled at least within the lumbar cord (Supplementary Fig. S7). To evaluate whether CS neurons make direct connections with lumbar MNs, we sacrificed the animals on P18. We found only few labeled cells in the cerebral cortex (Fig. 3a, 0 cells from 2 animals, 1 from 1, 2 from 1, and 3 from 1 animals). By contrast, a large number of transsynaptically labeled neurons were found in the brain stem and spinal cord (Fig. 3b–e; 166 ± 85 for brain stem, 846 ± 164 for spinal cord, n = 5), confirming that the monosynaptic tracing was itself successfully done (Fig. 3b–e and Supplementary Fig. S8). Thus few if any CS axons made direct synapses on the lower-leg MNs. These results suggest that CS neurons do not make promiscuous connections with MNs; instead, they show considerable preference to forelimb MNs.

### Direct CS-MN connections are eliminated during development

**Figure 4:**
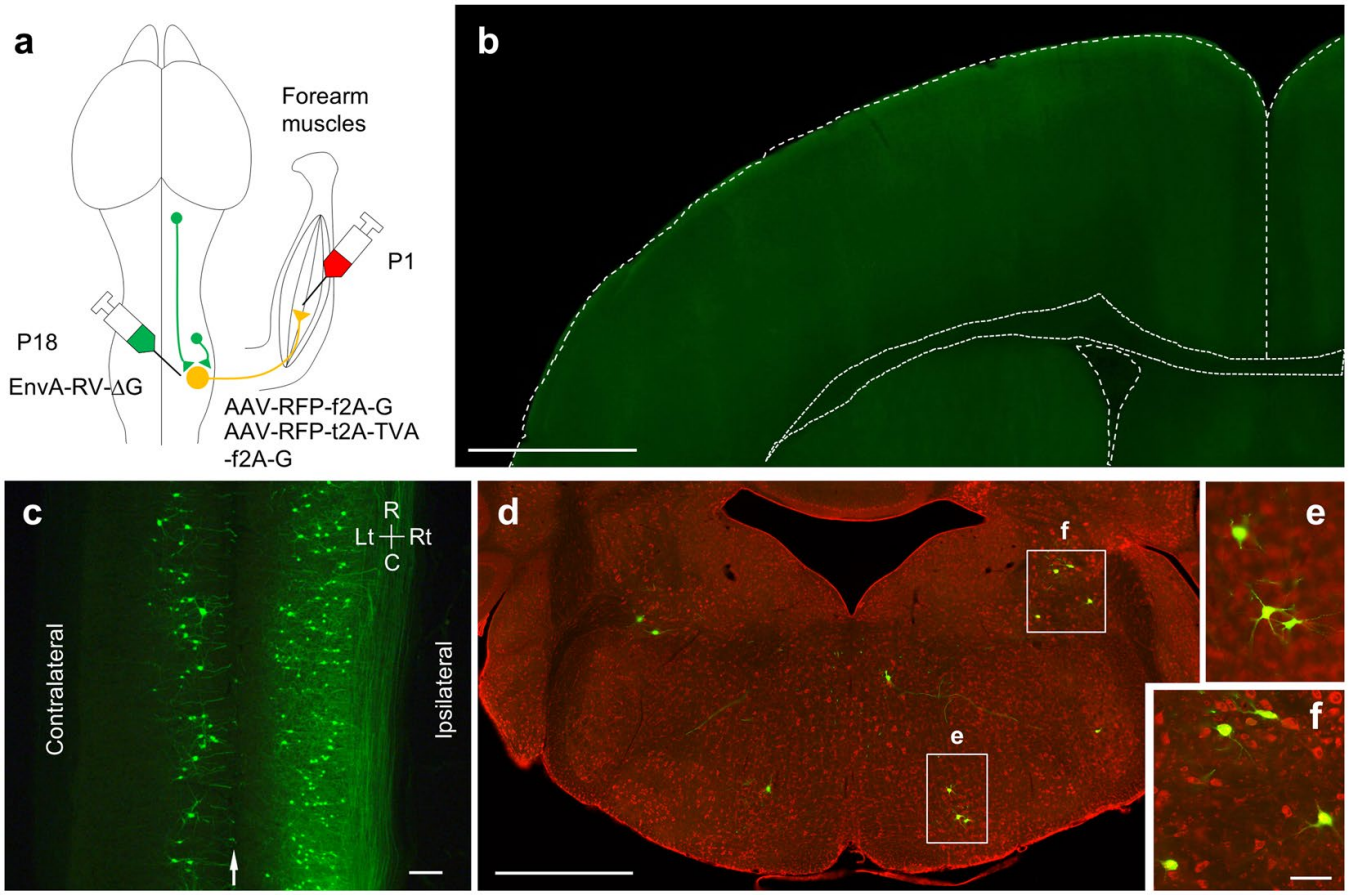
Monosynaptic tracing from forearm MNs in P26 animals. (**a**) Experimental design for the retrograde monosynaptic tracing with EnvA-RV-ΔG-GFP from P18 to P26. Intraspinal injections of EnvA-RV-ΔG-GFP on P18 targeted to forearm MNs expressing TVA, G protein and RFP after infection by AAVs through intramuscular injections on P1. (**b**) Representative view of the contralateral cortex on P26 showing the absence of CM cells. Image sequences from 10 coronal sections corresponding to the M1/S1 area were superimposed. Scale bar, 1 mm (**c**) Representative view of the spinal cord at C5 to Th1 (longitudinal section). C, caudal, R, rostral, Lt, left, Rt, right. Arrow, midline. Scale bar, 200 μm. (**d**) Example of a brain stem coronal section. The positions of GFP positive neurons are marked (upper). Red, a fluorescent Nissl staining by Neurotrace Red. Scale bar, 1 mm. (**e,f**) Higher power view of the selected regions in (**d)**. Scale bar 100 μm. The images are representative of five independent experiments.

**Figure 5:**
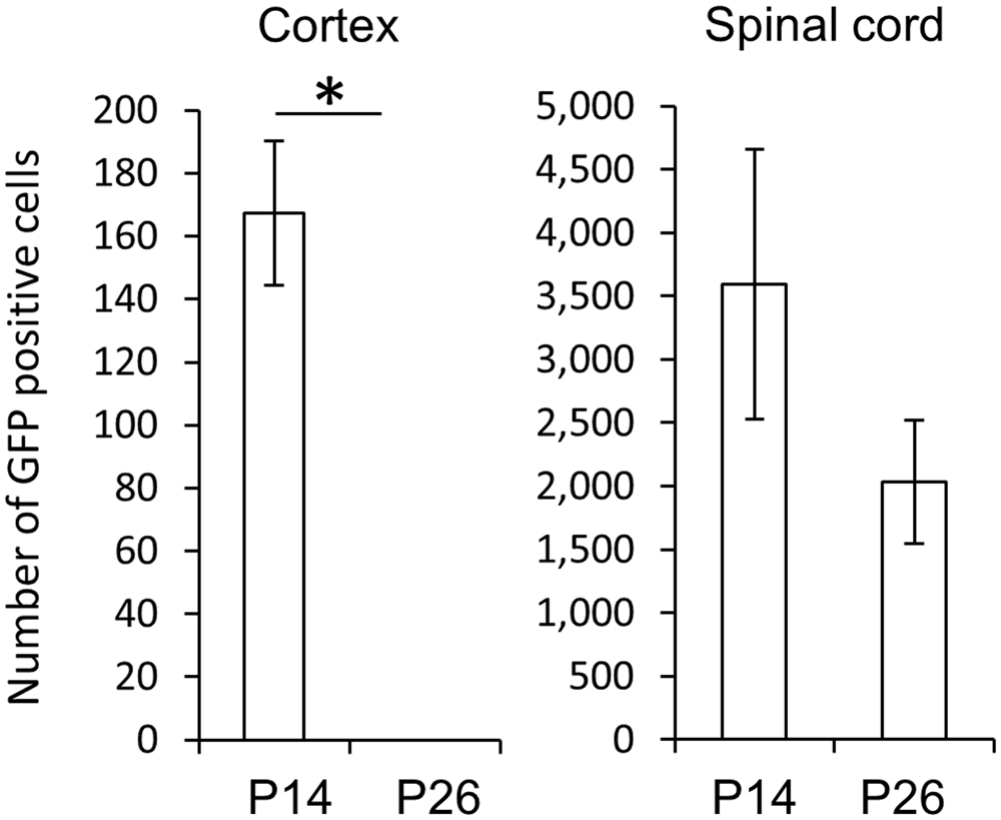
The direct CM connections were eliminated during development. Numbers of transsynaptically labeled neurons in the cortex and spinal cord at C5 to Th1 on P14 and P26. (n = 5 mice per group; data are represented as mean ± SEM. Man-Whitney U-test. *P* = 0.014 for cortex, *P* = 0.25 for spinal cord).

Finally, to address the question as to whether CS synapses on forearm MNs are maintained or eliminated after P14, we first injected RV-ΔG-GFP together with AAV expressing G protein into the forearm muscles on P22. However, we then found no GFP-expressing MNs in the cervical cord (n = 4). This result is consistent with the previous report that the efficiency of rabies infection from the neuromuscular junction greatly declines after P10^19^. As an alternative, we directly infected MN somata with rabies virus using a G protein-deficient rabies virus pseudotyped with EnvA. By itself, the EnvA-pseudotyped rabies virus cannot infect mouse neurons (Supplementary Fig. S9). The initial infection thus occurred only when the target neurons were engineered to express TVA^18,45^. To express TVAs selectively in forearm MNs, AAV encoding TVA was injected into the forearm muscles on P1. AAV encoding G protein was also co-injected for subsequent monosynaptic spread of the rabies virus. EnvA-RV-ΔG-GFP was injected into the cervical gray matter on P18 (Fig. 4a). Notably, despite labeling numerous cells in the cervical cord, we observed no labeled cells whatsoever in the cerebral cortex on P26, 8 days after the rabies virus injection (Fig. 4b,c; n = 5). To assess transsynaptic spread of the rabies virus from forearm MNs, the transsynaptically labeled neurons in the cervical cord were counted on P26. We found that the number of GFP-positive neurons on P26 was comparable to the number in animals examined on P14 (Fig. 5; 2030 ± 488 for P26 (n = 5) and 3591 ± 1064 for P14 (n = 5)), with similar distributions in the cervical cord (Fig. 4c). Numerous transsynaptically-labeled neurons were found in brain stem nuclei on P26 (189 ± 61, n = 5), including the red nucleus, vestibular nucleus and reticular nuclei (Fig. 4d–f and Supplementary Fig. S10). Because the GFP-positive neurons were found in the brain stem as early as 4 days after intraspinal injections (average 114.5, n = 2), we expect that the survival period (8 days) would be long enough to label the premotor neurons located in the cerebral cortex if they were present. These results suggest that direct CM connections are transient during development and so are absent in the adult.

## Discussion

The question whether direct CS-MN synapses are present only in higher primates or are present more widely in subprimate mammals, including rodents, has long awaited an answer. In a subprimate mammal, the mouse, the CS-MN connections are present at an early stage of development. Furthermore, the CS-MN connections would have been lost by P22 (see below), and so are absent in adult animals.

More importantly here we showed that as in higher primates, the CS-MN connections are with forearm MNs. In the macaque monkey, CM cells are distributed in the caudal part of M1 and area 3a, but not in the premotor region (area 6)^46^. It is suggested based on their connectivity that the rodent rostral forelimb area (RFA; M2) and caudal forelimb area (CFA), which are included in M1, are homologous to the primate premotor and primary motor (M1) areas, respectively^47^. CM cells in juvenile rodents are also distributed in M1, but there are almost none in M2. In addition, the somatosensory area has significant populations of CM cells in both juvenile rodents (S1) and primates (area 3a)^46^. These similarities may indicate an evolutionary connection between CM synapses in adult primates and juvenile rodents.

Our study revealed that CM cells preferentially make direct connections with forelimb MNs compared with the hindlimb MNs in postnatal mice during development. This pattern is similar to that of higher primates^48^, implicating evolutionary continuity.

Monosynaptic tracing from forearm MNs did not label cortical cells in P26 animals (Fig. 4). This was not due to failure of transsynaptic spread of the virus, as we found transsynaptically labeled neurons in the cervical cords of P26 animals in numbers comparable to those in P14 animals (Fig. 5). The survival period utilized to allow the rabies virus to spread could affect the number of transsynaptically labeled neurons distantly located from the MNs. But since the rubrospinal neurons, which are located in the midbrain, were labeled in our conditions, we think it should be long enough for the rabies virus to reach the cerebral cortex from the MNs in monosynaptic tracing on P26 from fore-arm MNs as well as on P18 from lower-leg MNs. Similarly, we would detect cortical inputs to lumbar MNs in juveniles on P18 that is 4 days after CS neurons make functional synapses onto lumbar neurons^43^ if they were present.

We used two types of recombinant rabies viruses: RV-ΔG-GFP for monosynaptic tracing from P6 to P14 and EnvA-RV-ΔG-GFP for tracing from P18 to P26. Both viruses carry the same genome but display different envelope glycoproteins: G protein and EnvA^49^. MNs infected with either virus generate a single type of infectious particle, RV-ΔG-GFP from G protein expressed by AAV. It is therefore highly unlikely that the two types of recombinant rabies viruses transsynaptically spread to different types of neurons. The efficiency of transsynaptic labeling from forearm MNs might also differ between P14 and P26. Nevertheless, because the number of transsynaptically labeled neurons in the cervical cord on P26 was comparable to that on P14 (Fig. 5), the absence of transsynaptically labeled cortical cells indicates developmental loss of direct CS-MN connections.

To estimate when CS-MN synapses are eliminated, it is necessary to know how long is the interval between injection of rabies virus targeted to starter neurons and the emergence of discernible transsynaptically-labeled presynaptic neurons. A study of transsynaptic labeling using the same type of rabies virus in the cerebral cortex showed that presynaptic neurons locally connected to pyramidal neurons targeted by the rabies virus were labeled on the fourth day after injection, but not on the second day^49^. We therefore estimate the delay period to be four days at the longest (P18-P22). Consequently, the CM synapses would have been eliminated by P22 (4 days after rabies virus injection into the spinal cord).

Direct CM synapses, during early postnatal life may play a role in the development of spinal circuits rather than in producing movement^50–52^. Since congenital absence of the CS projection causes the reduction of spinal MNs in number and the atrophy of the neuromuscular junctions^53^, the direct CM synapses may have a trophic support upon MNs. Alternatively, it is noteworthy that the direct CS-forearm MN connections are eliminated towards P22, which corresponds to the time of weaning. This transient neuronal connection could thus be involved in the forearm movements specifically needed at this stage of the animals’ life; for example, suckling behavior, which requires scanning and probing of the mother’s nipples. This activity may be replaced by the other types of probing movements that develop later, including whisker movement.

On P14, we abundantly found GFP-labeled neurons in the intermediate zone of the spinal cord including the lamina V, which is consistent with previous report^19,26^. These population innervates multiple motor neuron groups and proposed to regulate a set of MN activation patterns underlying various behavioral repertoire of animals^26^. Our finding that these premotor neurons in the lamina V persisted in P26 animals suggesting these types of circuits carry out their functions in more mature animals. This gives contrast to the CS neurons which transiently made synapses onto MNs.

Exuberant projection and connection in development are widespread across mammals^54,55^ and therefore were probably present in the mammalian ancestors. This study suggests that CM connection is one of exuberant connections in CS system in rodents and provides evidence that the CM connections have been eliminated in rodent group. On the other hand, the CM projection develops rapidly during first 5 months in macaque monkey^56^ and starts to innervate the gray matter around 30 weeks postconceptional age in man^57^ and are kept through their life. These phylogenic differences in CM connection could emerge through species-specific pruning of CS projection and connections in the common ancestor^52^, which might true for cortico-cortical connection^58^. It is tempting to speculate that these transient connections in subprimate mammals are primordia of the direct CS-MN connections seen in higher primates. To produce novel connections in adult neuronal circuits, it would be easier to leave previously transient and redundant connections than to create changes in the genome^12,59,60^. Further studies of the developmental changes in CS-MN connections in several representative mammalian species and the accompanying gene expression in MN and CS neurons will address this challenging question.

## Methods

### Animals

All animal procedures were performed in accordance with the National Institutes of Health Guide for the Care and Use of Laboratory Animals (eighth edition of National Institutes of Health) and were approved by the Ethics Committee of Teikyo University School of Medicine (No. 14-010). C57BL/6 mice of both sexes were used in monosynaptic tracing. We summarized animals used in this study (Supplementary Table 1).

### Acetylcholinesterase histochemistry

To visualize neuromuscular junctions in the forearm and lower-leg muscles, acetylcholinesterase histochemistry was carried out^23^. Animals were anesthetized with 4% isoflurane (Pfizer) and decapitated, after which the right forelimb and hindlimb were dissected. After removing the skin and fascia, the limbs were immersed in 4% paraformaldehyde (PFA) in 0.1 M phosphate buffer (PB) for 1 h at room temperature. The limbs were then washed with PB twice and immersed in 200 ml of PB containing 5 mM acetylthiocholine iodide, 40 mM glycine, and 13 mM copper sulfate for 4 h at 4 °C. After washing the limbs again for 2 min with PB, they were developed by immersion in 10% ammonium sulfide solution. The reaction was stopped by washing with PB.

### Virus preparation and retrograde monosynaptic tracing

Glycoprotein-deficient rabies virus (RV-ΔG-GFP) or avian sarcoma and leukosis virus (ASLV) (EnvA-RV-ΔG-GFP) expressing GFP were prepared as described previously (Mori and Morimoto 2014). We used two kinds of AAV to supply G protein and mCherry with a nuclear localized signal (RFP). TVA, the ASLV receptor^18^, was also encoded in one of the AAVs. One was prepared from pAAV-CAG-RFP-f2A-TVA-t2A-G-polyA and the other was from pAAV-CAG-RFP-f2A-G-WPRE-polyA. Both AAVs were produced as AAV serotype 6 in an adenovirus-free system using the three-plasmid cotransfection method and then purified using two sequential continuous CsCl gradients. For optogenetics, a plasmid vector encoding a variant of channelrhodopsin-2 (ChR2) fused to EYFP [pAAV-CaMKIIa-hChR2(H134R)-EYFP-WPRE-polyA] was a kind gift from Dr. Deisseroth (Stanford University, Stanford, CA). This AAV was produced as serotype 1.

For monosynaptic tracing beginning on P5-P6 or P22 from forearm MNs, and on P8 from lower-leg MNs, animals were anesthetized with 2% isoflurane. Incisions were made on the lateral and medial aspects of the limb skin to expose the forearm and lower-leg muscles. RV-ΔG-GFP (5.0 × 10^5^ infectious unit (IU)/ml), AAV6-CAG-RFP-f2A-G-polyA (8.0 × 10^6^ vector genome (vg)/ml) and 0.1% fast green FCF (Sigma) were mixed together and injected using a glass micropipette connected with Picopump (World Precision Instruments). A mixture of the viruses (0.54 μl) was injected into the forearm muscles, which included the extensor digitorum communis, extensor digitorum lateralis, extensor carpi ulnaris, flexor carpi radialis, palmaris longus, flexor digitorum superficialis, and flexor carpi ulnaris. The lower-leg muscles were injected: 1.9 μl into the gastrocnemius, 1.4 μl into the tibialis anterior and 0.5 μl into the soleus and extensor digitorum longus muscles. For control experiment, RV-ΔG-GFP was similarly injected described above without the AAV. The animals were sacrificed 8–9 (forearm) or 10 days (lower leg) after the injections. We injected viruses along the full length of the motor endplates as revealed by the whole limb acetylcholinesterase histochemistry described above. Injections were made at 1–3 points in small muscles or 5–8 points in large muscles. After the injections, the skin incision was closed with cyanoacrylate, and the animals were allowed to recover on a heat pad for 2 h before they were returned to the dam cage. For monosynaptic tracing in more mature animals (P18 to P26), viral suspension (360 nl) containing not only AAV6-CAG-RFP-f2A-TVA-t2A-G-polyA (1.9 × 10^7^ vg/ml), but AAV6-CAG-RFP-f2A-G-WPRE-polyA (1.9 × 10^6^ vg/ml) were required, probably because the former vector alone did not produce adequate amount of G protein. They were injected with 0.1% fast green on P1 into each muscle in the right forearm as described above. On P18, animals were anesthetized with isoflurane, and a laminectomy was performed to expose the dorsal surface of the spinal cord at C4-Th2. After incision of the dura and pia, 240 nl of the EnvA-RV-ΔG-GFP (4.9 × 10^5^ IU/ml) suspension were injected at seven points spaced every 0.5 mm along the right spinal cord (0.6 mm to the midline, 0.7 mm below surface of the spinal cord). For control experiment, EnvA-RV-ΔG-GFP were similarly injected without intramuscular injections of the AAVs. The skin was then closed, and the animals were allowed to recover on a heat pad for 2 h before returning them to the dam cage. For optogenetics, AAV encoding ChR2 (1.4 µl, 4.8 × 10^6^ vg/ml) was stereotaxically injected into the sensorimotor area of the left cortex on P0 using the following coordinates: 1.0 mm lateral, 1.5 mm anterior to lambda and 0.5 mm below the skin surface.

### Histology, immunofluorescence and imaging

Animals were deeply anesthetized with 4% isoflurane and perfused first with PB containing heparin (60 mg/l) and then with 4% PFA in 0.1 M PB. The brain and the spinal cord were then removed and post-fixed with the same fixative at 4 °C overnight. Brains were coronally sectioned at 50 μm thickness using a Microslicer (Dosaka EM) and counterstained with Neurotrace Red (Life Technologies). Spinal cords were transversely or longitudinally sectioned at 50 μm with the microslicer. Fluorescent images were taken using a Nanozoomer (Hamamatsu Photonics) equipped with a 20 × objective lens (NA, 0.75) or Nikon epifluorescent microscope equipped with a 4 × objective lens (NA, 0.13).

For immunofluorescent labeling, free-floating sections (50 μm) were permeabilized with 0.5% Triton X-100 in PBS and blocked with blocking buffer (5% goat serum, 0.1% Triton X-100 in PBS pH 7.4). Primary antibodies diluted in blocking buffer were incubated over-night at room temperature. After washing with PBS containing 0.1% Triton X-100, secondary antibodies diluted in blocking buffer with or without addition of Neurotrace Red to visualize laminar distribution of the cortex were over-night at room temperature. Sections were then washed and mounted using 90% glycerol containing 0.1% sodium azide. Antibodies used in this study were rat anti-CTIP2 antibody (1:500, abcam), goat anti-Choline acetyl transferase antibody (1:500, Millipore), Goat anti-rat IgG antibody coupled to Cy5 (1:300, IBL) and Donkey anti-goat IgG antibody coupled to Cy5 (1:300, IBL). Immunofluorescent imaging was carried out on a confocal-laser scanning microscope (Nikon A1) with 10 × objective lens (NA, 0.45) or Nikon epifluorescent microscope equipped with a 10 × objective lens (NA, 0.3).

### Data analysis and quantification

Images acquired using the Nanozoomer were down-sampled (corresponding to 5 × images) and exported as tiff files. Image sequences for each single section were z-projected to a single image using ImageJ. For the cortex, we used Photoshop (Adobe) to manually align the z-projections to reconstruct a series of coronal brain sections. We set the midline of the cerebral hemispheres of each section as zero for the mediolateral axis (x axis). We manually marked the positions of GFP-positive pyramidal neurons using ImageJ with the “Cell Counter” plugin. X coordinates and the section number were used to prepare the distribution map. For quantification of the labeled neurons in the brainstem and spinal cord, we used images taken with Nanozoomer or the Nikon epifluorescent microscope and performed the same procedure described above, except omitting alignment of the z-projections of the brainstem or spinal sections from single animals. We counted the number of GFP-positive, RFP-negative cells in the spinal cord from C5 to Th1 for forearm injections and from L1 to L5 for lower-leg injections. Cell counting was carried out by individuals who were blind to the experimental groups. For the brain stem, we excluded the red nuclei from the quantitative analysis. To compare the distribution of CM cells and C7-projecting CS neurons, we re-used the original data of cortical map previously published in which the retrograde tracer spreads to >80% of the gray matter in dorso-ventral axis to cover the entire anterior horn and intermediate zone of the spinal cord where the somata and dendrites are located^43^. This map was obtained by injection into single segment (C7) with retrobeads but is essentially similar to one obtained by injection into broader cervical region (C3 to C7) with retrobeads^61^. We divided the cortical area into three regions to separate three loci of CM distribution (Fig. 2a). The boundaries were normalized to the cortical outline and overlain on the distribution map of the C7-projecting CS neurons. We counted the number of CM cells or CS neurons in the secondary motor (M2), the primary sensorimotor (M1/S1) and secondary somatosensory (S2) areas to calculate their proportions. In Fig. 2b, the boundary between M1 and S1 areas was taken from the Allen brain atlas, normalized to the cortical outline, and overlaid on the M1/S1 areas of the CM map.

### Retrograde labeling of cervical MNs

Retrograde labeling of cervical MNs with cholera toxin subunit B-conjugated Alexa Fluor 488 (CTB-Alexa 488, Life Technologies) was carried out according to a standard procedure described previously^12,29–34^. On P6-P7, mice were anesthetized using 2.8% isoflurane. To retrogradely label forearm MN pools in the cervical spinal cord, we used a 10-µl Hamilton syringe fitted with a 27 G needle (Terumo) to inject forearm muscles with CTB-Alexa 488 diluted to 1 mg/ml in PBS. CTB-Alexa 488 is taken up from the axons and transported to and accumulated in the MN somata. Restriction of CTB-labeled neurons to the MNs is based on the fact that MNs are only neurons in the spinal cord extending the axons to the skeletal muscles.

### Photoactivation and whole-cell recording

Transverse slices (400 µm thick) of spinal cord at C7–8 from P8-P10 mice were prepared at 4 °C using chilled cutting solution (in mM: 234.0 sucrose, 2.5 KCl, 1.25 NaH_2_PO_4_, 10.0 MgSO_4_, 0.5 CaCl_2_, 26.0 NaHCO_3_, 11.0 glucose) and then incubated in artificial cerebrospinal fluid (ACSF) (in mM: 119.0 NaCl, 2.5 KCl, 1.0 NaH_2_PO_4_, 2.5 CaCl_2_, 1.3 MgCl_2_, 26.0 NaHCO_3_, 20.0 glucose) for at least 45 min at room temperature. The solutions were continuously bubbled with a mixture of 95% O_2_ and 5% CO_2_. To photoactivate ChR2-expressing CS axons, light from a blue LED (465 nm, LEX2-B, Brainvision) controlled by a stimulator (SEN-7203, Nihon Kohden) was guided into a 0.5-mm optical fiber (Edmund Optics Japan) positioned with a micromanipulator such that the power density of the LED light was 8.9 mW/mm^2^ at the slice surface. Stimulus frequencies were 0.1 or 0.05 Hz. To inhibit polysynaptic responses, high concentrations of divalent cations (7 mM Ca^2+^, 3 mM Mg^2+^) in ACSF were perfused for at least 20 min before photoactivation of ChR2. Whole-cell patch recordings were made from spinal neurons at room temperature. Patch electrodes were filled with internal solution (in mM: 128 cesium gluconate, 20 CsCl, 10.0 HEPES, 0.2 EGTA, 2.0 ATP (Mg^2+^ salt) and 0.2 GTP (Na^+^ salt), adjusted to pH 7.2 by CsOH) containing 1% dextran rhodamine B (Life Technologies) and had resistances of 6–12 MΩ. The membrane potential was clamped at −90 mV to record EPSCs. Latency was measured from the end of photostimulation. Cells with series resistances >40 MΩ were excluded from our analysis. In addition, at the end of the recordings in the current-clamp mode, we confirmed that action potentials were generated upon injection of depolarizing current. Before and after the recordings, fluorescent images were collected using a cooled CCD camera system (MiCAM02; Brainvision).

### Statistical Methods

All data are presented as the mean ± standard error of the mean (SEM). The proportions of CM cells and C7-projecting CS neurons were compared using Student’s t-test. Group sample sizes were chosen based on previous studies. The numbers of transsynaptically labeled neurons in the cervical cord were compared between P14 and P26 using Man-Whitney U-test.

## Electronic supplementary material

Supplementary information

## Data Availability

The datasets generated during and/or analyzed during the current study are available from the corresponding author on reasonable request.
