# Supplementary material for: Higher primate-like direct corticomotoneuronal connections are transiently formed in a juvenile subprimate mammal
Source: Sci Rep. 2018 Nov 8;8:16536. doi: 10.1038/s41598-018-34961-z (PMC6224497; doi:10.1038/s41598-018-34961-z)
Supplement: Supplementary file 1 — Supplementary information [file 41598_2018_34961_MOESM1_ESM.pdf]

Scientific Reports
Supplementary information

Higher primate-like direct corticomotoneuronal connections are transiently formed in a juvenile subprimate mammal

Authors: Naoyuki Murabe[1], Takuma Mori[2, 6], Satoshi Fukuda[1], Noriko Isoo[1], Takae Ohno[1], Hiroaki Mizukami[3], Keiya Ozawa[3, 4], Yumiko Yoshimura[2, 5] and Masaki Sakurai [1*]

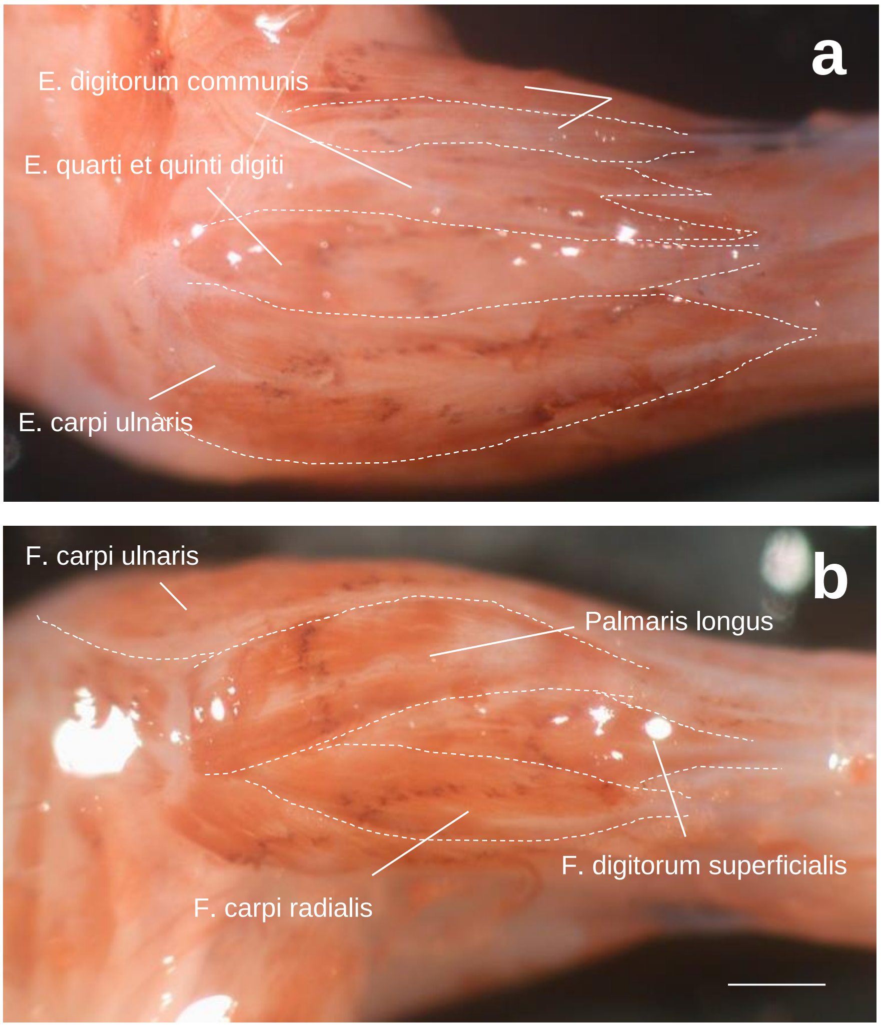

Supplementary Fig. S1. Distribution of the neuromuscular junctions in the forearm muscles. Neuromuscular junctions in the lateral (**a**) and medial (**b**) aspects of the right forearm of P5 mice visualized using acetylcholinesterase histochemistry. Scale bar, 1 mm.

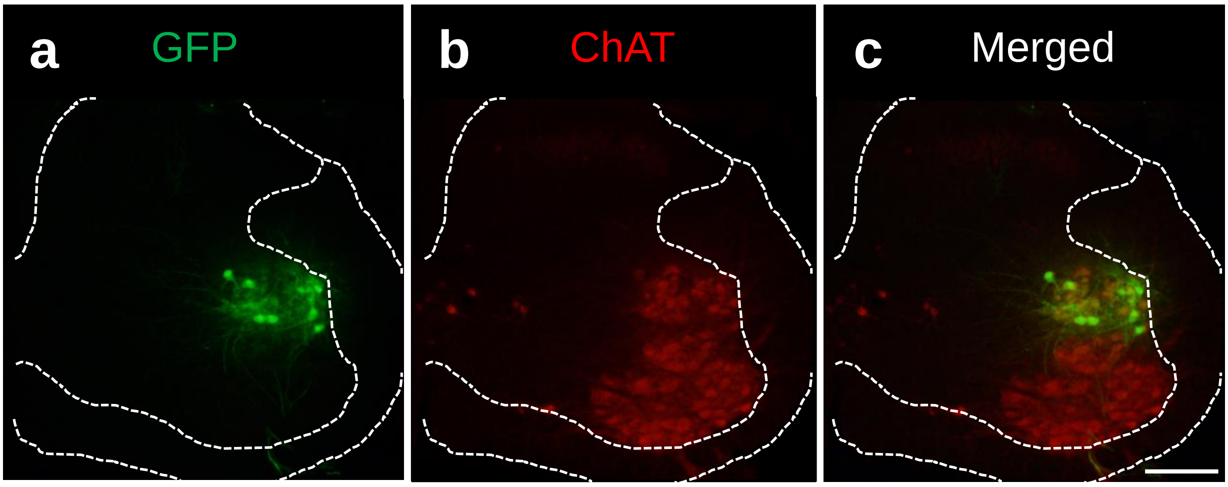

Supplementary Fig. S2. Injection of RV-ΔG-GFP alone into the forearm muscles on P7 labeled only the forearm MNs on P14.

(a) Retrogradely labeled GFP-positive cells, (**b**) Anti-choline acetyl transferase (ChAT) immunolabeling showing MNs, (**c**) Merged image. Bar, 200 μm.

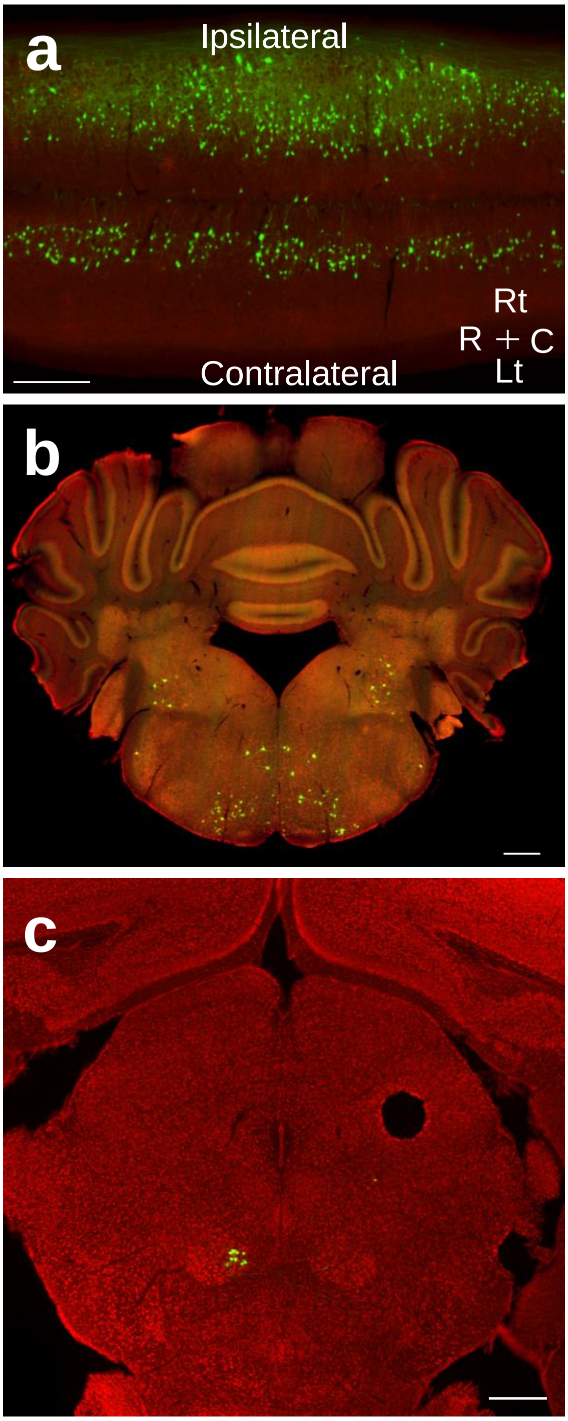

Supplementary Fig. S3. Monosynaptic tracing from forearm MNs showing the GFP-labeled neurons in the cervical spinal cord and brain stem.

(**a**) Example of a horizontal section of cervical spinal cord showing transsynaptically-labeled neurons. Examples of a coronal sections in the medulla oblongata (**b**) and red nucleus (**c**) showing transsynaptically labeled-neurons after monosynaptic tracing. Animals were received intramuscular injection of RV-ΔG-GFP and AAV6-RFP-f2A-G on P5-6 and sacrificed on P14. Red, a fluorescent Nissl staining by Neurotrace Red (**b**, **c**). Scale bar, 500 μm. C, caudal, R, rostral, Lt, left, Rt, right.

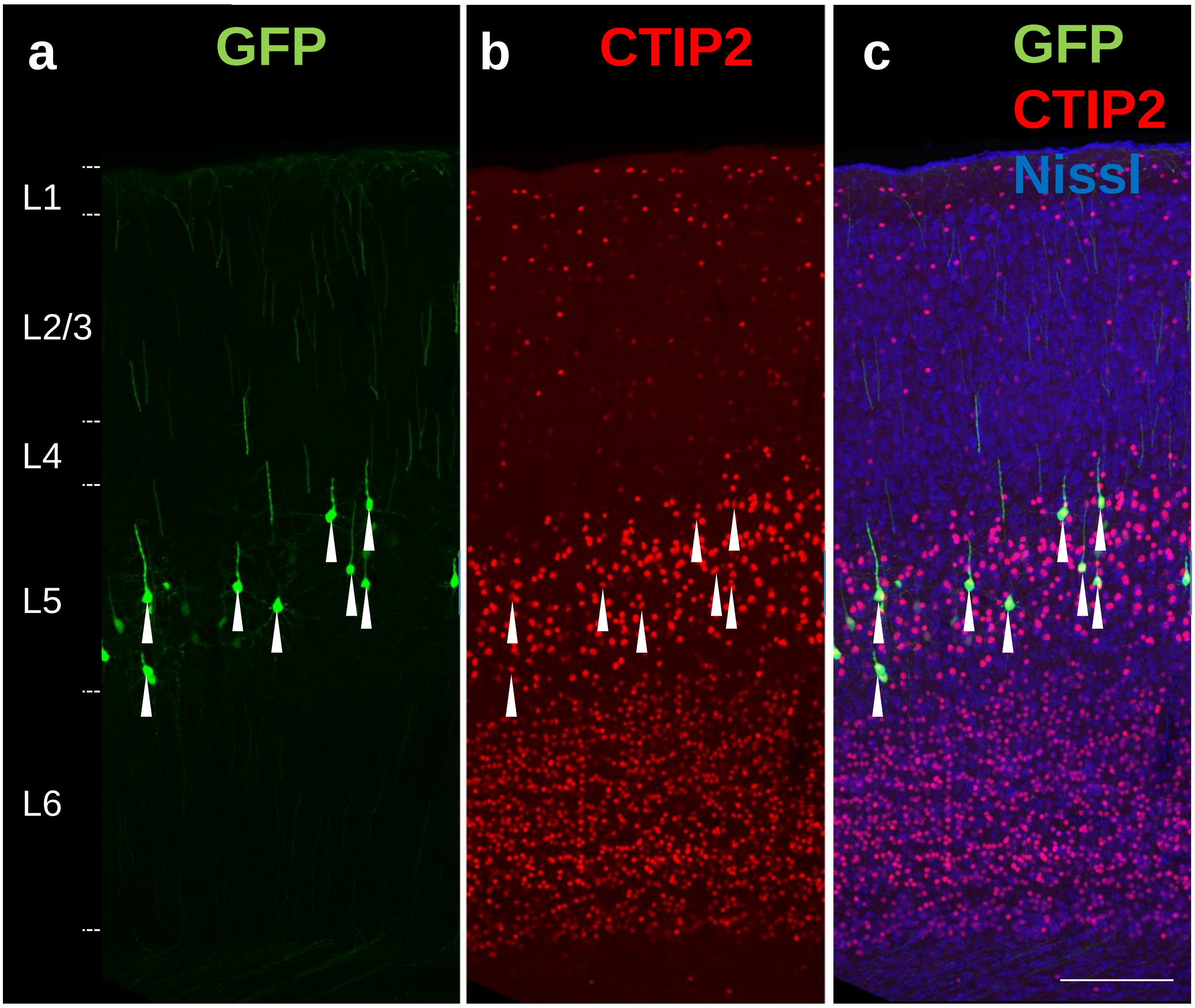

Supplementary Fig. S4. Identities of the cortical premotor neurons.

GFP-labeled neurons in P14 cortex after monosynaptic tracing from forearm MNs located in the layer V (**a**), all of which expressed CTIP2 that is preferentialy expressed in the corticospinal neurons in the layer V (**b**). (**c**) Merged image with Neurotrace Red staining (blue, fluorescent Nissl staining) showing laminar structure of the cortex. Arrowheads, examples of double positive cells. Bar, 200 μm.

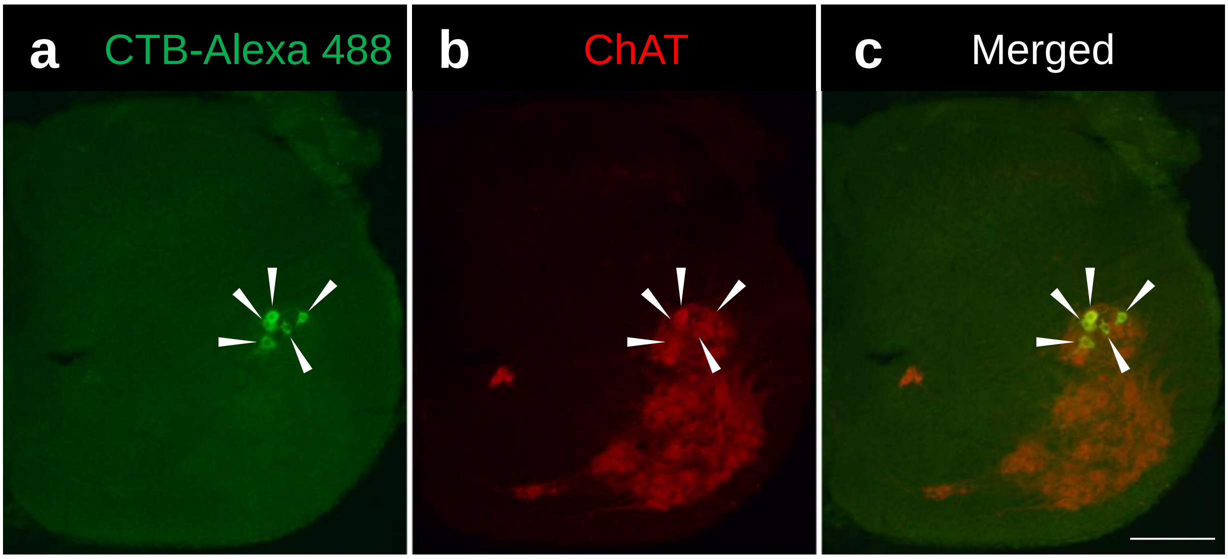

Supplementary Fig. S5. Retrogradely labeled cells with CTB-Alexa 488 from the forearm muscles were all ChAT-positive in the cervical cord.

(**a**) CTB-Alexa 488, (**b**) Anti-ChAT immunolabeling showing MNs, (**c**) Merged image. Arrowheads, CTB-Alexa 488 labeled cells. Bar, 200 μm.

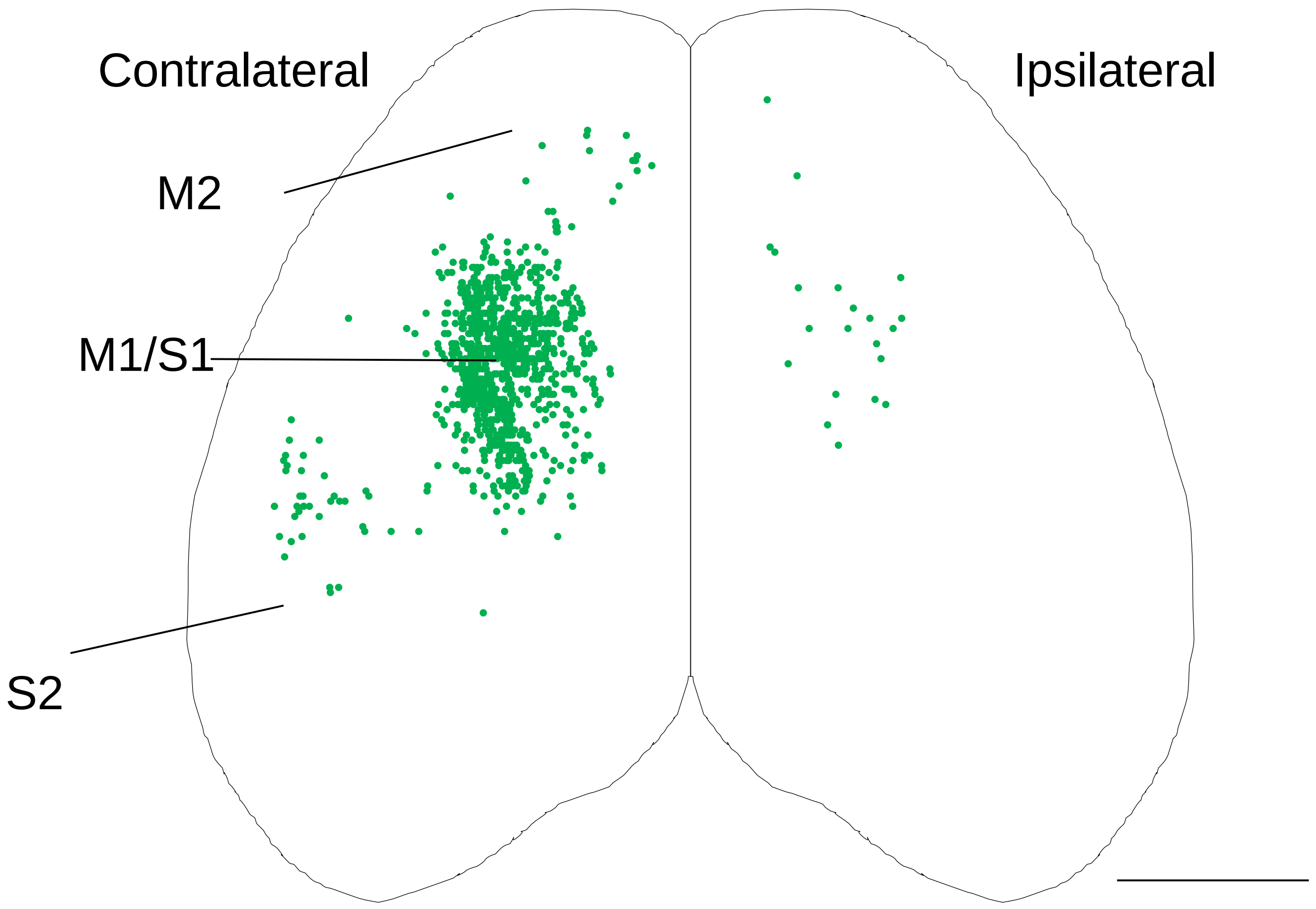

Supplementary Fig. S6. Distribution of CM cells in the contralateral and ipsilateral hemispheres of the cerebral cortices of early juveniles.

Data from 5 animals are plotted. Scale bar, 2 mm

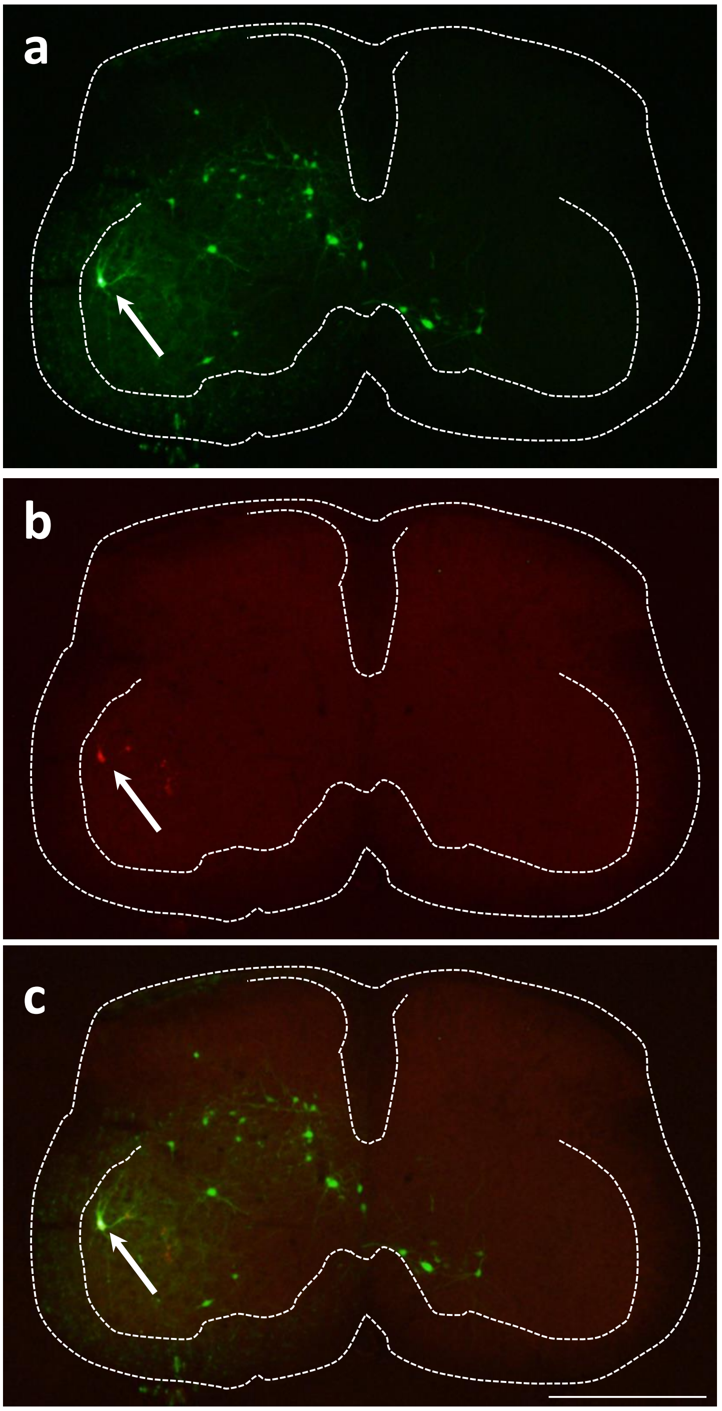

Supplementary Fig. S7. Monosynaptic tracing from lower-leg muscle MNs. Lumbar (L4) spinal cord 6 days after intramuscular injections of RV-ΔG-GFP and AAV6-RFP-ΔG showing GFP^+ neurons (**a**), a RFP^+ neuron indicative of a starter MN (arrow) (**b**). (**c**) Merged image. GFP^+ and RFP^- neurons were transsynaptically labeled. Outline of the spinal cord and gray matter were shown in dotted line. Scale bar, 500 μm. Animals were received intramuscular injection of RV-ΔG-GFP and AAV6-RFP-f2A-G on P8 and sacrificed on P14.

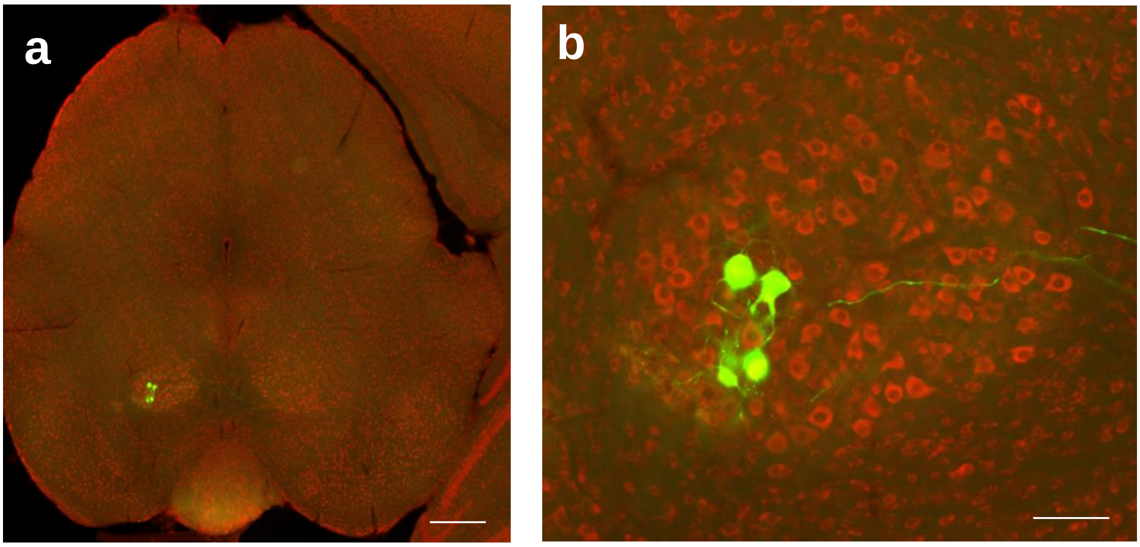

Supplementary Fig. S8. Example of transsynaptically labeled-neurons in the midbrain from the lower-leg MNs.

(**a**) Contralateral red nucleus containing transsynaptically labeled neurons. (**b**) High power view of the contralateral red nucleus. Red, a fluorescent Nissl staining by Neurotrace Red. Animals were received intramuscular injection of RV-ΔG-GFP and AAV6-RFP-f2A-G on P8 and sacrificed on P18.Scale bars indicate 500 μm in **a** and 100 μm in **b**, respectively.

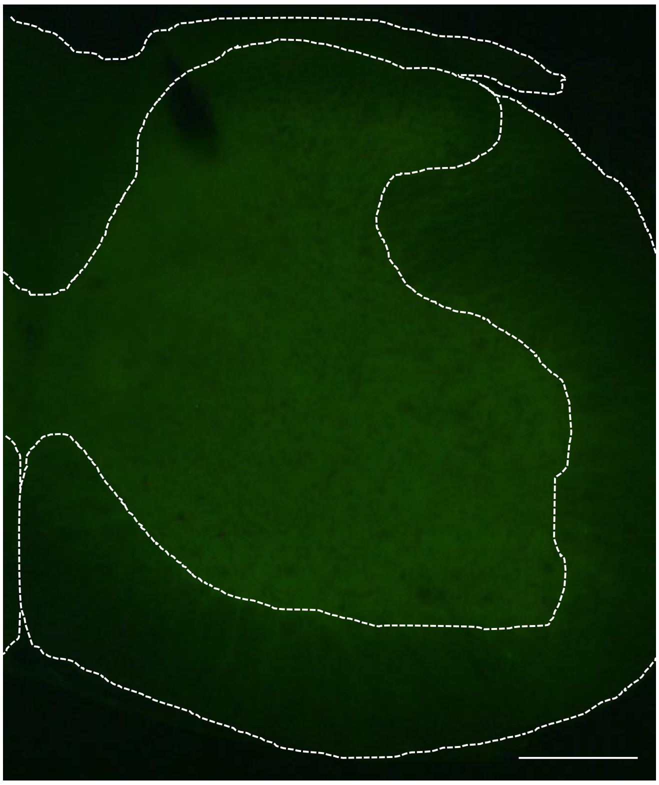

Supplementary Fig. S9. EnvA-pseudotyped rabies virus cannot infect spinal cord neurons in mice.

Intraspinal injection of EnvA-RV-ΔG-GFP alone (without intramuscular injections of AAVs expressing TVA and G protein) produced no GFP-labeled cells in the cervical cord. EnvA-RV-ΔG-GFP was injected on P18 and animals were sacrificed on P22. Bar, 200 μm.

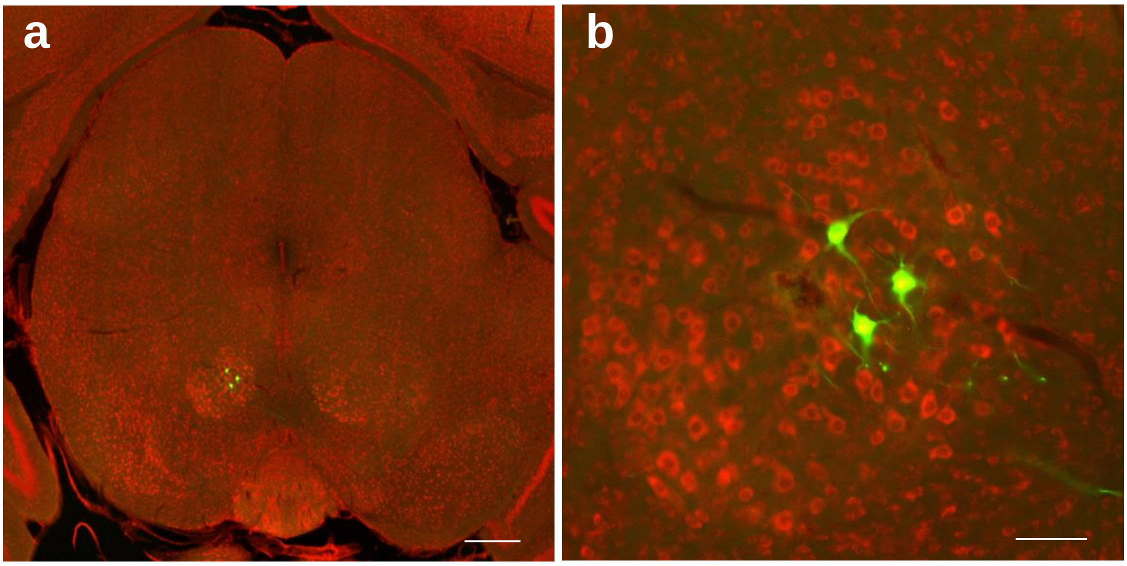

Supplementary Fig. S10. Transsynaptically labeled neurons from forearm MNs in the contralateral red nucleus of a P26 mouse.

(**a**) Contralateral red nucleus containing transsynaptically labeled neurons on P26. (**b**) High power view of the labeled cells. Red, a fluorescent Nissl staining by Neurotrace Red. Scale bars indicate 500 μm in **a** and 100 μm in **b**, respectively. Animals were received intramuscular injection of AAVs expressing TVA, G protein and RFP on P1, and intraspinal injection of envA-RV-ΔG-GFP on P18 and sacrificed on P26.

Supplementary Table S1. Experimental animals

Exps	AAV injection(s)		Rabies injection		Survival time[1] (days)	Age for fixation	The number of animals
	Age	Site	Age	Site			
Choline esterase histochemistry							
	-	-	-	-	-	P2	1
	-	-	-	-	-	P4	1
	-	-	-	-	-	P6	1
	-	-	-	-	-	P7	7
	-	-	-	-	-	P10	2
	-	-	-	-	-	P14	1
	-	-	-	-	-	P49	1
Monosynaptic tracing							
	P5	FM[2]	P5	FM	9	P14	3
	P6	FM	P6	FM	8	P14	2
	P7	FM	P7	FM	7	P14	3
	P8	LM[3]	P8	LM	6	P14	2
	P8	LM	P8	LM	10	P18	5
	P22	FM	P22	C5-Th1[4]	6	P28	4
	P1	FM	P18	C5-Th1	4	P22	2
	P1	FM	P18	C5-Th1	8	P26	5
Control experiments related to monosynaptic tracing							
	-	-	P7	FM	7	P14	3
	-	-	P18	C5-Th1	4	P22	3

[1]Period from rabies injection to fixation

[2]forearm muscles

[3]lower-leg muscles

[4]Spinal cord between the fifth cervical and first thoracic segments
